# Advances in the Immune Regulatory Role of Non-Coding RNAs (miRNAs and lncRNAs) in Insect-Pathogen Interactions

**DOI:** 10.3389/fimmu.2022.856457

**Published:** 2022-04-06

**Authors:** Ulrich Aymard Ekomi Moure, Tingshan Tan, Lin Sha, Xiaoqin Lu, Zhi Shao, Guang Yang, Yi Wang, Hongjuan Cui

**Affiliations:** ^1^Affiliated Hospital of Southwest University, the Ninth People’s Hospital of Chongqing, Chongqing, China; ^2^Medical Research Institute, Southwest University, Chongqing, China; ^3^Department of Gastrointestinal Surgery, the Ninth People’s Hospital of Chongqing, Chongqing, China; ^4^State Key Laboratory of Silkworm Genome Biology, Key Laboratory of Sericultural Biology and Genetic Breeding, Ministry of Agriculture, Southwest University, Chongqing, China

**Keywords:** insect immune pathways, insect–pathogen interaction, miRNAs and lncRNAs, mRNA targets, immune modulation

## Abstract

Insects are by far the most abundant and diverse living organisms on earth and are frequently prone to microbial attacks. In other to counteract and overcome microbial invasions, insects have in an evolutionary way conserved and developed immune defense mechanisms such as Toll, immune deficiency (Imd), and JAK/STAT signaling pathways leading to the expression of antimicrobial peptides. These pathways have accessory immune effector mechanisms, such as phagocytosis, encapsulation, melanization, nodulation, RNA interference (RNAi), lysis, autophagy, and apoptosis. However, pathogens evolved strategies that circumvent host immune response following infections, which may have helped insects further sophisticate their immune response mechanisms. The involvement of ncRNAs in insect immunity is undeniable, and several excellent studies or reviews have investigated and described their roles in various insects. However, the functional analyses of ncRNAs in insects upon pathogen attacks are not exhaustive as novel ncRNAs are being increasingly discovered in those organisms. This article gives an overview of the main insect signaling pathways and effector mechanisms activated by pathogen invaders and summarizes the latest findings of the immune modulation role of both insect- and pathogen-encoded ncRNAs, especially miRNAs and lncRNAs during insect–pathogen crosstalk.

## 1 Introduction

**Figure 1:**
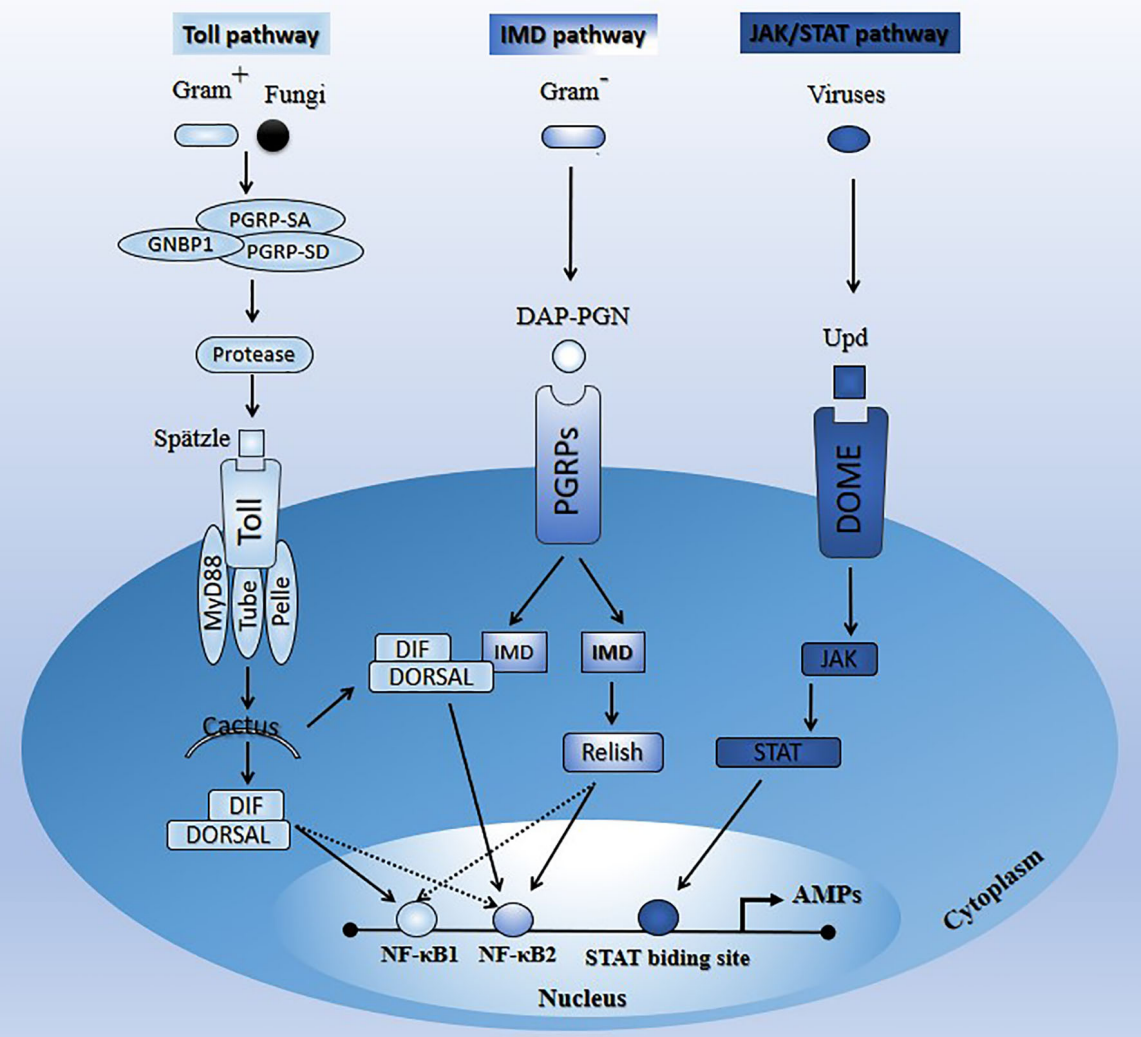
Schematic illustration of the main signaling pathways (Toll, Imd, and JAK/STAT) in insects upon microbial attacks. See Section 2 for the description of the paths.

Insects are often subjected to pathogen (bacteria, fungi, viruses, etc.) attacks, and their survival indicates advanced defense mechanisms (1). These organisms of interest endue physical barriers preventing intruders from entering their body cavity (hemocoel) (2, 3). These intruders generally reach insect body parts through the degradation of the cuticle by enzymatic digestion or by ingestion (midgut) (4), which are followed by the induction of the host’s immune defense mechanisms *via* the binding of their pathogen-associated molecular patterns (PAMPs) (5). Activation and coordination of insects’ innate immune defenses rely on evolutionary and highly conserved immune factors. This immunity is classified into humoral and cellular reactions (6). Humoral responses include the production of antimicrobial peptides (AMPs) (7). Studies showed that the Toll, immune deficiency (Imd), and the Janus kinase/signal transducers and activators of transcription (JAK/STAT) pathways are the most characterized in insects, and their activation results in the production of effector molecules such as AMPs with the potential to kill insect invaders (8–12) (Section 2; **Figure 1**). However, it was reported that these signaling pathways could be encountered by pathogens and lead to their replication and proliferation (13–15). Cellular reactions, meanwhile, rely on insect hemocytes (primary immune cell defenses) involved in phagocytosis, nodulation, encapsulation, autophagy, apoptosis, and so on (4, 16, 17). Given all this arsenal of immune defense described above, insects lack an adaptive immune response, unlike mammals, and rely only on innate immunity (18, 19). We may speculate that insect immune defense against pathogens lacks a memory immunity that allows rapid and robust responses to neutralize and kill pathogens quickly. Nonetheless, we are far from reaching the exhaustive portrayal of how these organisms that represent the Earth’s most abundant and diverse living organisms defend themselves.

Several factors such as insect gut microbiota, nutritional stress, and noncoding RNAs (ncRNAs) play an immune-modulatory role during insect–pathogen crosstalk (20–23). Noncoding RNAs (ncRNAs), also termed nonprotein-coding RNAs are RNA molecules that do not have the potentiality to encode proteins (24). In addition to ribosomal RNA (rRNA) and transfer RNA (tRNA), ncRNAs can be divided into two groups, small ncRNA (sncRNA) and long ncRNA (lncRNA), based on their size. sncRNA can be further divided into miRNA, piRNA, siRNA, etc. lncRNA can be subdivided into sense, antisense, intronic, intergenic, *cis*-, and *trans*-RNA based on their biogenesis and mechanism of action (25). This review concentrates on the involvement of miRNAs and lncRNAs in insect immune regulation upon pathogen invasions. miRNAs and lncRNAs have varied regulatory roles at the epigenetic, transcriptional, or posttranscriptional levels and participate in almost all biological processes, including immunity and host–microbial interactions. Moreover, lncRNAs and miRNAs showed differential expression levels upon insect pathogenic infections or pesticide exposure, further proving their involvement in modulating insect immune responses. For instance, infection of *Galleria mellonella* (*G. mellonella*) with both uropathogenic *Escherichia coli* (UPEC) and asymptomatic bacteriuria (ABU) caused significant changes in the abundance of miRNAs in the larvae. It highlighted the differential expression of 147 conserved miRNAs and 95 novel miRNA candidates. In addition, by utilizing next-generation sequencing (NGS), Dubey et al. (26) identified 126 miRNAs from *Aedes aegypti* (*A. aegypti*) cell line Aag2, and 13 of them were regulated during chikungunya virus (CHIKV) infection. Moreover and interestingly, the review written by Awais et al. (27) summarizes the diversity and multitude of differentially regulated miRNAs in *Bombyx mori* (*B. mori*) upon several viral attacks, such as *B. mori* cypovirus (BmCPV), *B. mori* nucleopolyhedrovirus (BmNPV), and *B. mori Autographa californica multiple* nucleopolyhedrovirus (AcMNPV). In parallel, the *Drosophila melanogaster* (*D. melanogaster*) lncRNA CR44404 (renamed lincRNA-IBIN) was highly induced (1300-fold) by Gram-positive or Gram-negative bacteria in *Drosophila* adults or a parasitoid wasp *Leptopilina boulardi* in *Drosophila* larvae (28). Moreover, lncRNA-155 targeted the protein tyrosine phosphatase 1B to modulate innate immunity against influenza A virus (IAV) infection in mice (29). In contrast, the lncRNA-1317 supported host antiviral defense mechanism during dengue virus serotype 2 (DENV-2) infection in *A. aegypti* (30). Although the fact that the expression levels of insect-encoded miRNAs and lncRNAs are altered during microbial attacks, little is known about the different mechanisms employed by these ncRNAs to regulate insects' immune defenses in response to those invaders.

This review focuses on two types of ncRNAs, lncRNAs and miRNAs. It summarizes the main immune signaling pathways and immune effector mechanisms engaged upon microbial invasions and reports the latest findings supporting the involvement of insect- and pathogen-derived miRNAs and lncRNAs in the immunological insights related to insect–pathogen interactions. It lastly provides perspectives on gaps and where future investigation must be oriented.

## 2 Insect Main Signaling Pathways Triggered by Pathogens

### 2.1 The Toll Signaling Pathway

This insects’ pathway is responsive to Gram-positive bacteria and fungi and largely controls the expression of AMPs (28–30). The Toll pathway is a NF-κB-associated signaling pathway (8, 9). Stimulation of insects’ Toll pathway by appropriate pathogens induces proteolytic cascades activated by secreted recognition molecules, such as peptidoglycan receptor proteins (PGRP-SA, PGRP-SD, GNBP1) (11, 31–34). These recognition molecules are highly evolutionarily conserved from insects to mammals. For instance, *Drosophila*, mosquito, and mammals have families of 13, 7, and 4 PGRP genes, respectively (35). In *Drosophila* where the molecular components of the Toll signaling pathway and their functions in immune response against pathogens are well characterized (36–38), the receptor membrane Toll is activated and dimerized by the mature proteolytic product Spätzle (39–42), which subsequently causes the recruitment of three intracellular death domain-containing proteins, MyD88, Tube, and Pelle (43–45). The IκB homolog Cactus is then phosphorylated and degraded by the proteasome, leading to the release of members of the nuclear factor NF-κB family (Dif or dorsal) to translocate to the nucleus (46–48), and activate genes encoding potent antifungal and antibacterial peptides (AMPs), such as Drosomycin (*Dr*) (11, 38, 49). The Toll-dorsal pathway in *Drosophila* and the interleukin-1 receptor (IL-1R)-NF-kB pathway in mammals are homologous signal transduction pathways that mediate several different biological responses, including immunity (50).

### 2.2 The Imd Signaling Pathway

This pathway is well characterized in *Dipterans* such as *Drosophila*; however, in *Hemipterans* like aphids and assassin bugs, several components of that pathway are lacking. Imd signaling components appear to be absent in *Rhodnius prolixus* (*Reduviidae*) and *Acyrthosiphon pisum* (*Aphididae*) (51–53). However, some study reported that the Toll and Imd pathways are present in the hemipteran lineages like stinkburg *Plautia stali*, but their functionality is blurred (54). The Imd pathway is mainly responsive to Gram-negative bacteria and controls the synthesis of several AMPs (55, 56).

During the infection, insect Imd transmembrane receptor proteins (PGRPs) recognize pathogen diaminopimelic acid-peptidoglycan (DAP-PGN). This recognition induces a cascade reaction resulting in Relish (NF-κB family member) activation (cytoplasm) and translocation (nucleus) for AMP production (*Diptericin*) (38, 57). Like the Toll pathway, the Imd pathway is a NF-κB-associated signaling pathway (8, 9). Although the Imd and Toll pathways have independent functions and mediate the specificity of innate immune responses towards different microorganisms, some AMP genes can be activated by both pathways. Interestingly, Tanji et al. (58) showed that a synergic interaction occurs between the Toll and Imd signaling pathways, downstream of the latter. Specifically, upon signal stimulation, Dif, dorsal, and relish factors can independently bind to NF-κB1 or NF-κB2, and relish interacts after that with Dif or dorsal to synergistically promote transcription. Alternatively, signal stimulation may enhance the formation of relish/Dif or relish/dorsal heterodimers and bind preferentially to NF-κB2 to regulate transcription synergistically with interaction with NF-κB1 (58).

### 2.3 The JAT/STAT Signaling Pathway

The JAK/STAT pathway was originally identified as a cytokine signaling pathway in mammals (59–62). This pathway provides an antiviral protection (27, 28, 63). Unlike the Toll and Imd pathways, little is known about the transcriptional cascade induced by the JAK/STAT pathway (14, 64). Souza-Neto et al. (65) showed that the JAK-STAT pathway is part of the *A. aegypti* mosquito’s antidengue defense and may act independently of the Toll pathway and the RNAi-mediated antiviral defenses.

The comparative genomic analysis of *A. aegypti*, *Anopheles gambiae*, and *D. melanogaster* genome sequence revealed orthologs for the core JAK/STAT pathway components (dome, Hop, and STAT) (66). Activation of this pathway in *Drosophila* starts when the virus-induced extracellular cytokine unpaired (Upd) binds to the cellular receptor dome. The latter recruits STAT, which then dimerizes and translocates to the nucleus and activates the transcription of AMP genes (nitric oxide synthase) after binding to the STAT-binding site (12, 65). Protein inhibitor of activated STAT (PIAS) and suppressor of cytokine signaling (SOCS), are two negative regulators of the JAK-STAT pathway in *D. melanogaster* (67). Orthologs of these two regulators (SUMO and SOCS) have been identified, confirming the proximity and evolutionary conservation of this pathway between mammals and insects (65).

## 3 Insect Immune Effector Mechanisms

**Figure 2:**
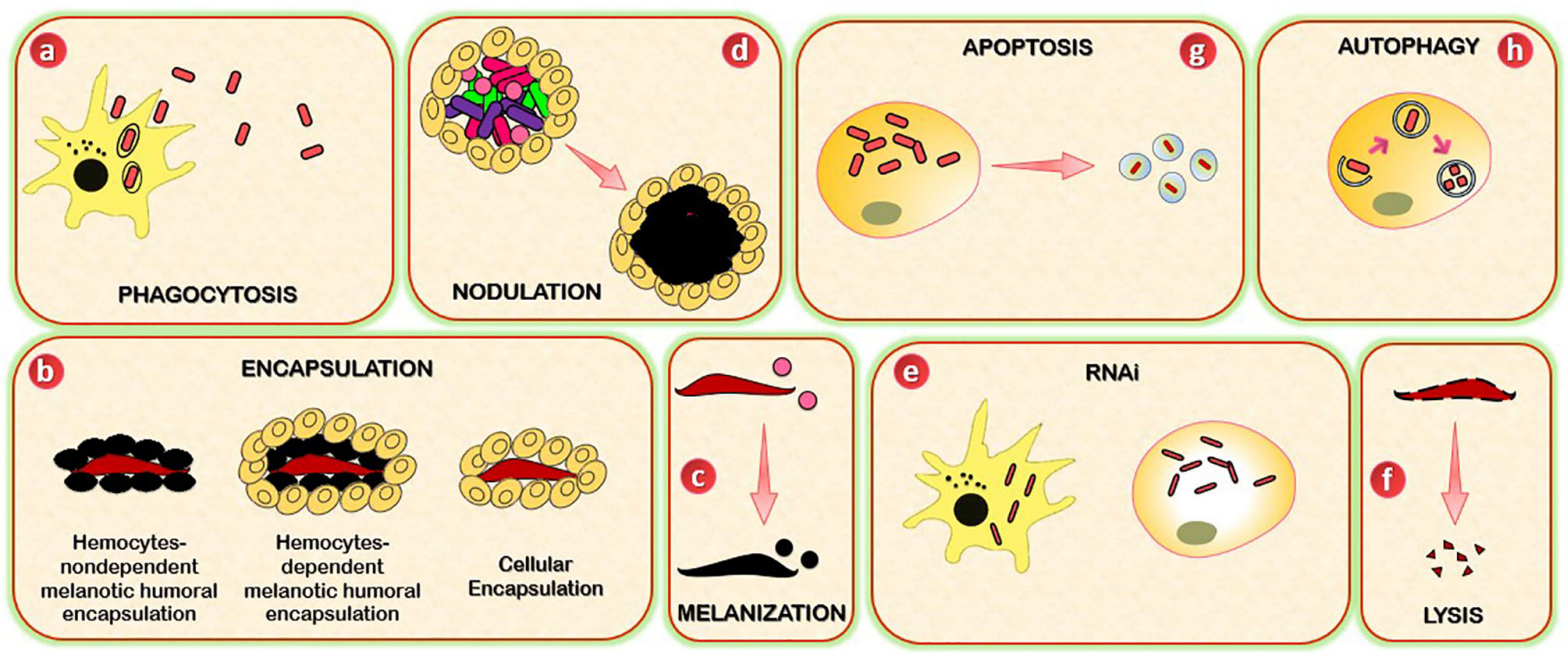
Schematic representation of insects’ immune effector mechanisms. **(A)** Insects use phagocytosis to neutralize and kill small pathogens. This process is mediated by phagocytes (hemocytes or granulocytes). **(B)** Encapsulation (cellular and melanotic) is a defense mechanism that insects used when pathogens are too large to be phagocytosed. Cellular encapsulation occurs without melanization, whereas melanotic humoral encapsulation is dependent on PO activity and can occur with or without the assistance of hemocytes. **(C)** Melanization is a process based on the conversion of PPO to PO, which leads to the formation of the melanotic capsule (melanotic enzymes) which mediates the killing of the foreign agent. **(D)** Nodulation is a process by which immune cells (granulocytes) adhere to each other to create layers that surround many bacteria or fungal spores. The granulocytes release their contents, which trap the bacteria in a flocculent material. This step is often followed by melanization. **(E)** RNAi mechanism is based on the ribonuclease cleavage of viral dsRNA and is specifically used against viruses, and can be mediated by immune circulating cells (hemocytes, macrophages). **(F)** Lysis is a mechanism through which insects kill pathogens by disrupting their cellular membrane. The process involves the participation of peptides and proteins with antimicrobial activity, including lysosomes. **(G)** Autophagy provides protection to insects against pathogens through the degradation of cytoplasmic material (bacteria or viruses). **(H)** Apoptosis is a programmed cell death mediated by caspases. The killing of pathogens includes the formation of apoptotic bodies.

**Figure 3:**
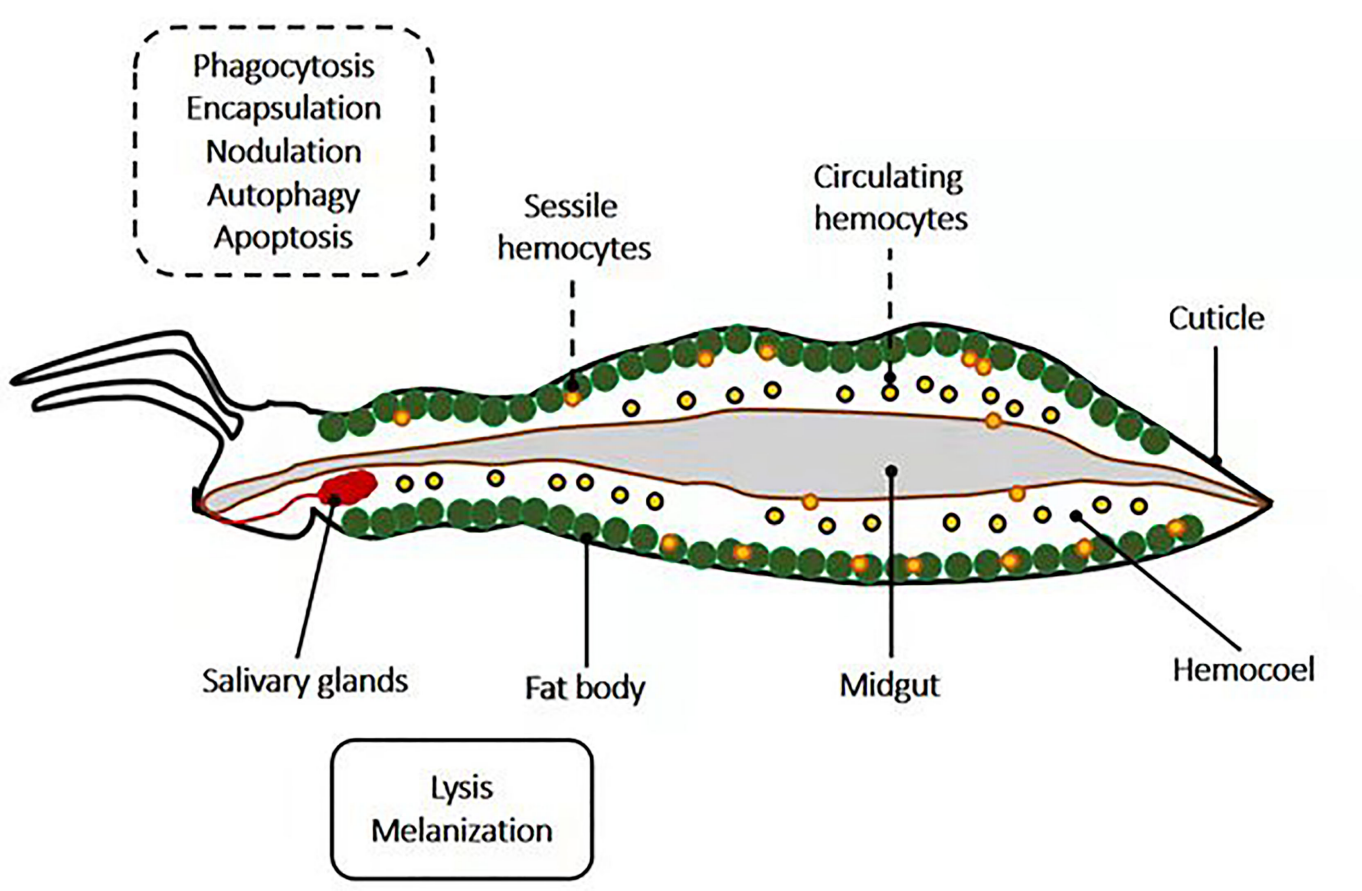
Schematic representation of the localization of insects’ immune effector mechanisms. Mechanisms such as phagocytosis, encapsulation, nodulation, autophagy, and apoptosis occur in insect hemocytes (sessile and circulating), which are located in the hemocoel. Meanwhile, insect parts such as salivary glands, the fat body, and midgut are potent sites for lysis and melanization.

Pathogen neutralization and death are accomplished *via* insect immune effector mechanisms, known to be interconnected and to work synergistically (**Figure 2**). The primary immune cells are the hemocytes. Hemocytes are found in circulation (circulating hemocytes) and attached to tissues (sessile hemocytes), where they phagocytose, encapsulate and nodulate pathogens, and produce humoral immune factors. The fat body, the midgut, the salivary glands, and other tissues produce numerous humoral immune factors with, among other things, lytic and melanizing activity (**Figure 3**).

### 3.1 Phagocytosis

Small pathogens that become melanized, such as bacteria, are often phagocytosed (68, 69). Phagocytosis is an evolutionarily conserved cellular immune process used by vertebrates and invertebrate animals to neutralize and kill small pathogens. Due to its incredible speed, it takes seconds to hydrolyze foreign bodies following their internalization (69–71), and hundreds of bacteria can be phagocytosed at any time (72). The phagocytes, which can be circulating and sessile hemocytes (73) or granulocytes in the cases of *Lepidoptera*, *Hemiptera*, and mosquitoes, and the plasmatocytes of fruit flies, identify the foreign body. The latter is then internalized into a membrane-delimited phagosome, which then fuses with a lysosome for enzymatic hydrolysis-mediated degradation (68, 69, 73, 74).

Although the intracellular patterns regulating phagocytosis remain poorly understood, phagocytosis initiates by binding of a cell-surface PRR or a humoral PRR on a PAMP. PRRs that have been empirically shown to be involved in phagocytosis include thioester-containing proteins, Nimrod proteins, DSCAM, β-integrins, and PGRPs (75–79). Different PRRs have different specificities. For example, *D. melanogaster* PGRP-LC mediates the phagocytosis of *Escherichia coli* (*E. coli*), but not *Staphylococcus aureus* (*S. aureus*) (79), and NimC1 mediates the phagocytosis of *S. aureus* and, to a lesser extent, *E. coli* (76).

### 3.2 Encapsulation

Encapsulation is a cellular immune response that insects use in response to pathogens that are too large to be phagocytosed. Two types of encapsulation are described in insects: cellular encapsulation, mainly described in *Lepidoptera*, and melanotic humoral encapsulation, more typical in some dipterans like *Drosophila* (80). Cellular encapsulation can occur without any sign of melanization. In contrast, melanotic encapsulation relies on PO activity and can occur with or without the assistance of hemocytes. In cellular encapsulation, granulocytes and plasmatocytes of *Lepidoptera*, plasmatocytes, and lamellocytes of *Drosophila* are the main hemocyte types involved in encapsulation (81, 82). In *Lepidoptera*, an inner layer of granulocytes and an outer one of plasmatocytes surrounded encapsulated objects (83, 84). Insects such as dipteran and lepidopteran larvae commonly employed this response to infection with the eggs of parasitoid wasps. In *Lepidoptera*, encapsulation starts when granulocytes attach to form a layer of cells, in a process dependent on the binding of integrins to specific sites defined by an Arg-Gly-Asp (RGD) sequence (85). This layer of cells surrounding the pathogen is in turn covered by several layers of plasmatocytes, which are then circled by the adhesion of additional granulocytes. A similar process occurs in *Drosophila*, except that the cells involved are plasmatocytes and lamellocytes (86). Depending on the pathogen and the insect, the capsule may become melanized.

### 3.3 Melanization

Melanization is an enzymatic process used by insects in several mechanisms, including immunity. The process involves coordinating pattern recognition receptors, serine proteases, serine protease inhibitors, and melanin production enzymes. When PRRs (β-1,3 glucan recognition proteins, C-type lectins, and Gram-negative binding proteins) identify PAMPs (87–89), the serine protease cascade is induced and leads to the conversion of the pro-phenoloxidase (PPO) to phenoloxidase (PO) (90, 91), which finally culminates in the formation of melanotic capsules. The killing of the foreign agent is mediated by two factors: the pathogen’s surrounding by the proteinaceous capsule and the oxidative stress damage or starvation (92–94). In addition, melanization also assists in the clearing of dead or dying pathogens (95, 96). Many of the enzymes and PRRs that drive the melanization response are produced by hemocytes, with oenocytoids (crystal cells) being the primary producers of PPO (68, 97). Melanization is another essential facet of the mosquito immune defense against fungi infection. It plays a crucial role in encapsulating and retarding invasive *Bauveria bassiana* (*B. bassiana*) growth and dissemination in mosquitoes (98).

### 3.4 Nodulation

Although the molecular patterns underlining this immune process are still poorly understood, nodulation relies on eicosanoid-based signaling and the extracellular matrix-like protein, Noduler (99, 100). This process starts with the adherence of granulocytes to each other to create layers that surround many bacteria or fungal spores. The granulocytes release their contents, which trap the bacteria in a flocculent material. Plasmatocytes then aggregate around the surface of the nodule. This step is often followed by melanization (101, 102).

### 3.5 RNAi

RNA interference (RNAi) is an RNA-based mechanism for gene silencing. RNAi could be used to manage insect pests (103, 104). Nevertheless, one of the natural functions of RNAi is to protect the organism from viral infection (105–108).

The small interfering RNA (siRNA) pathway is the primary RNAi path that participates in the insect response against the virus. The process involves the ribonuclease cleavage of viral double-stranded RNA (dsRNA) by dicer-2 (Dcr2), forming a complex with its cofactor R2D2. This cleavage culminates in the production of viral-derived siRNAs (~21 nucleotides), and these siRNAs are loaded into pre-RNA induced silencing complexes (RISC) that include Argonaute-2 (Ago2). The siRNA in a RISC complex is unwound, one strand is discarded, and the other binds complementary viral RNA, which triggers its destruction by Ago2. The binding of viral RNA by Dcr2 also activates the transcription of Vago, which is a cysteine-rich polypeptide that negatively controls virus replication (109). In mosquitoes, the transcriptional activation of Vago is mediated by an NF-κB transcription factor associated with the Imd pathway (Rel2), and in turn, Vago activates the JAK/STAT pathway (110). The PIWI-associated RNA pathway (piRNA) also acts in the RNAi-mediated antiviral defense of mosquitoes but in a manner independent of dicer (105, 106).

### 3.6 Lysis

Pathogen killing *via* lysis refers to death due to the immune-based disruption of the cellular membrane. Some of the earliest studied insect factors that induce pathogen death *via* lysis are AMPs (~2 and 20 kDa). These peptides and proteins were initially identified for their antimicrobial activity *in vitro* (111–113). Most AMPs can be grouped into four categories (114–116). The defensin and defensin-like peptides (defensin, drosomycin, holitricin, sapecin, and others) are rich in cysteines and contain cysteine disulfide bonds that have activity against Gram-positive bacteria. Still, others combat Gram-negative bacteria and fungi. The cecropin and cecropin-like peptides (moricins) are α-helical peptides that have activity against Gram-positive and Gram-negative bacteria and fungi. The attacins and gloverins are glycine-rich peptides and are mainly active against Gram-positive bacteria, whereas the gloverins, have activity against Gram-positive bacteria, Gram-negative bacteria, or fungi. The lebocins (lebocin, drosocin, metchnikowin, and others) are proline-rich peptides and are active against Gram-positive bacteria, Gram-negative bacteria, and some fungi. The production of antimicrobial peptides is governed by immune signaling pathways, as seen above with Toll, Imd, and JAK/STAT pathways.

Lysozymes are another family of proteins with lytic activity. Lysozymes hydrolyze the β-1,4-glycosidic linkage between *N*-acetylmuramic acid and *N*-acetylglucosamine of the peptidoglycan present in bacteria’s cell wall. Lysozymes are usually present in low, constitutive levels and are transcriptionally upregulated following infection. Although lysozymes are classically active in the lytic antibacterial response (117, 118), some also have antiplasmodium and antifungal activity (119, 120) and can activate the PO-based melanization pathway (121, 122).

### 3.7 Autophagy

Autophagy is an evolutionarily conserved mechanism involved in the degradation of cytoplasmic material through the lysosomal degradation pathway. It plays crucial roles in cellular homeostasis, adaptive response to nutrient deprivation, energy homeostasis, and survival during starvation (123, 124). In immunity, autophagy provides insect protective immune responses against both viral and bacterial attacks. Evidence showed that the Toll pathway induces autophagy during an antiviral response. It is excluded in the activation of autophagy during an antibacterial response and therefore needs further investigation (125). In *Drosophila*, the activation of autophagy by vesicular stomatitis virus (VSV) is mediated by Toll-7 recognition of VSV-G, a glycoprotein on the virion surface. This PAMP-PRR interaction leads to autophagy initiation by acting on the (PI3K)-Akt-signaling pathway (126, 127). The connection of a Toll receptor to antiviral autophagy in flies suggests an evolutionarily conserved requirement for PRR in triggering autophagy between insects and mammals. Autophagy may, in some cases, promote virus infection as shown for the Sindbis virus, where the activation of the (PI3K)-Akt-signaling pathway enhances the infection of insect cells (128). *Lysteria monocytogenes* invades and replicates in hemocytes and is recognized by PGRP-LE (a PRR which senses a DAP-type PGN), eliciting an autophagic response that prevents intracellular growth of the bacterium and promotes host survival (125).

### 3.8 Apoptosis

Apoptosis is a form of programmed cell death. The caspase, Dronc, and the adaptor protein, Ark, form a complex at the molecular level. Dronc activates effector caspases such as Drice and Dcp1, and these caspases cleave proteins in a manner that eventually leads to programmed cell death (129). In *Lepidoptera*, apoptosis is involved in the response against baculoviruses (130). In mosquitoes, this process appears to be involved in the response against West Nile Virus and Sindbis virus (131, 132), and in the fruit fly, apoptosis protects against *Drosophila* C virus in a manner that is dependent on the phagocytosis of virus-infected apoptotic cells (133).

## 4 Role of ncRNAs in Insect Immunity Upon Pathogenic Invasions

Using high-throughput sequencing techniques and advanced bioinformatics tools, researchers have done great work in discovering and identifying novel insect ncRNAs and their regulated transcripts, respectively (134, 135). The involvement of ncRNAs in insects’ immunity is evidenced, and changes in expression levels of these insect elements generally occur to be relatively altered upon microbial attacks. Below, we go beyond the altered differential expression of these insect elements and highlight their immune targets and influence on insect immune responses to pathogen invasions. Inversely, we will also consider the regulation of insect immunity by pathogen-encoded ncRNAs. **Tables 1** and **2** enclose both conserved and novel insect- and pathogen-derived miRNAs and lncRNAs and their targets in insect-pathogen crosstalk.

**Table 1:** Novel and conserved insect-derived miRNAs and their immune targets.

Insect	miRNA	Target gene	Expression level	Host target	Ref.
*G. mellonella*	gme-new-135-3p	*TNF-8-like*	–	*G. mellonella*	(136)
	gme-miR-274-3p	*TNF-8-like*	–		
	gme-miR-8-5p	*TNF-8-like*	–		
	gme-new-161-3p	*Poly(A)-pol*	–		
	gme-new-160-5p	*zf-LITAF-like*	Up		
	gme-new-135-5p	*Invertebrate-type lysozyme*	–		
	gme-miR-263a-5p	*Invertebrate-type lysozyme*	–		
	gme-new-70-3p	*Linear gramicidin synthase subunit D*	–		
	gme-new-147-3p	*Long-chain-fatty-acid CoA ligase 5*	–		
	gme-new-54-3p	*Ras guanine-nucleotide exchange factor*	–		
	gme-new-40-3p	*phosphatidylinositol 3-kinase*	Up		
	gme-new-138-3p	*AMP-dependent synthetase/ligase*	Up		
	gme-new-72-3p	*Atlastin*	–		
	gme-new-4-5p	*Long-chain-fatty-acid-CoA ligase 4*	–		
	gme-new-135-3p	*Long-chain-fatty-acid-CoA ligase ACSBG2*	–		
*Drosophila*	miR-959	*Tube*	Down	*Drosophila*	(137)
	miR-960	*Tube*	Down		
	miR-961	*Dorsal*	Down		
	miR-962	*Dorsal*	Down		
	miR-962	*Toll*	Down		
	let-7	*sec2p*	Down	*B. bassiana*	(23)
	miR-100	*C6TF*	Down		
*A. aegypti*	miR-2b	*URM*	Down	*A. aegypti*	(26)

**Table 2:** Novel and conserved insect-derived lncRNAs and their immune targets.

	LncRNA	Target gene	Expression level	Host target	Ref.
*L. striatellus*	MSTRG15394	*PI*	Up	*L. striatellus*	(138)
	MSTRG31066	*PI*	Up		
	MSTRG31416	*PI*	Up		
	MSTRG3494	*CREB-A*	Up		
	MSTRG12639	*CREB-A*	Up		
	MSTRG21101	*CREB-A*	Up		
	MSTRG32119	*CREB-A*	Up		
	MSTRG33257	*CREB-A*	Up		

### 4.1 Role of miRNAs in Insect Immunity During Microbial Invasions

**Figure 4:**
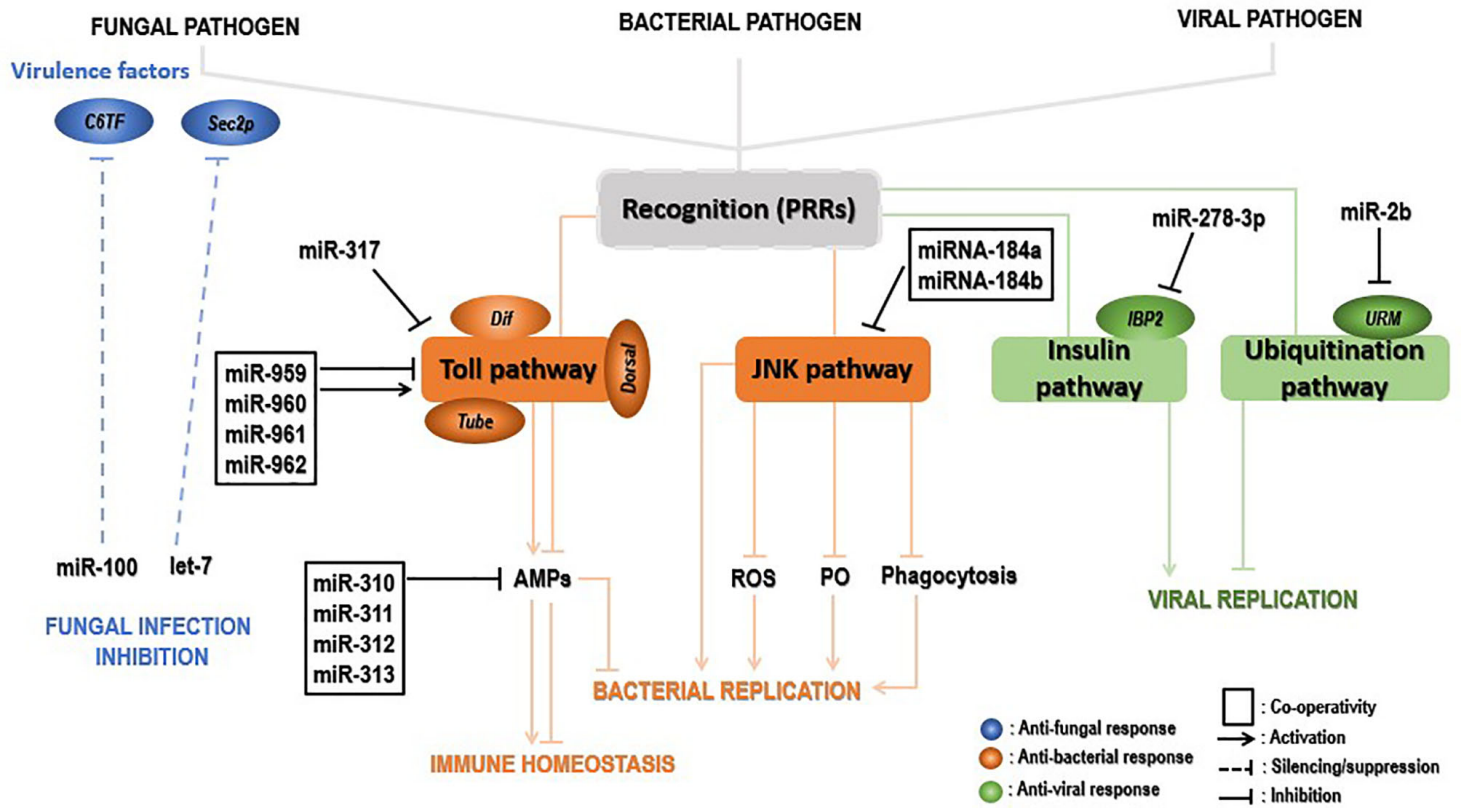
Insect immune defense modulation by miRNAs upon pathogenic invaders. Upon fungal invasion, insect miRNAs can silence fungal virulence genes, such as *C6TF* and *Sec2p*, and inhibits its replication (blue). Upon bacterial invasion, insect miRNAs individually or synergistically regulate the Toll pathway key components (*Tube*, *Dorsal*, *Dif*, etc.) positively, leading to activation of AMP gene effectors and inhibition of bacterial replication. Those elements can negatively modulate the same signaling at the late stage of the infection, decreasing the AMP gene expression for insect immune homeostasis. On the other hand, insect miRNAs can promote bacterial replication by inhibiting the JNK signaling pathway and indirectly those under its control (ROS, PO, phagocytosis) (in the pea aphid *Acyrthosiphon pisum*, for instance) (brown). Lastly, IBP2 is significantly upregulated upon viral invasion of insects. The latter’s expression is drastically repressed by insect miRNAs, leading to viral replication. In contrast, insect miRNAs inhibit viral replication by downregulating components (*URM*) of the ubiquitinylation process (green).

miRNAs play a crucial role in host–pathogen interactions. The dynamic miRNA–mRNA is essential for immune response to pathogen attacks, including regulating critical insect signaling pathways to promote or inhibit innate immune responses and maintain homeostasis. For instance, differentially expressed miRNAs and mRNAs represented extensively dynamic changes during *Drosophila* with *Micrococcus luteus* (*M. luteus*) infection and enriched diverse signaling pathways, including Toll and Imd as other signaling pathways (139). **Figure 4** summarizes the immune regulatory role of insect miRNAs when invaded by pathogens.

#### 4.1.1 Upon Fungal Invasion

The expression levels of miRNAs in the insect–pathogen crosstalk used to be altered. Detection and characterization of miRNA cellular targets would therefore provide more insights to understand the immune modulatory role of these miRNAs. Insect immune mechanisms may deal directly with the pathogens by eliminating them from the host organism or disarm them by abolishing the synthesis of toxins and virulence factors that promote the invasion and destructive action of the invader within the host. Insect-encoded miRNAs seem to preferentially deal with fungal pathogens by disarming them through the suppression of invaders’ virulence factors. This silencing interferes with some translation factors associated with 5’-cap to 3’-tail structures of mRNAs (140). Recently, a study revealed mosquitoes increase the expression levels of both let-7 and miR-100 miRNAs when the fungus *B. bassiana* penetrates inside their hemocoel. Both miRNAs translocate into the fungal hyphae to silence the virulence-related genes *sec2p* and *C6TF*, encoding a Rab guanine nucleotide exchange factor and Zn(II)_2_Cys_6_ transcription factors, respectively (23). Suggestions state that extracellular vesicles (EVs) may transport those insect miRNAs to the fungal pathogen. To our knowledge, this is the first report describing the cross-kingdom miRNA transfer from arthropod hosts to their pathogen cells. Globally, the above evidence uncovers an insect defense mechanism whereby infection leads to the upregulation of specific miRNAs that are transferred to the invading fungus and suppress fungal virulence genes, in this way, bestowing antifungal protection. This study opens avenues to improve fungal virulence by expression of host miRNA sponges.

#### 4.1.2 Upon Bacterial Invasion

Another crucial role played by miRNAs is to restore insects’ immune homeostasis by negatively regulating immune signaling pathways. This role is achieved by an individual or synergistic action of miRNAs, which repress expression levels of hosts’ immune signaling key components. *Dl* and *Toll* are critical transcription and transmembrane factors, respectively, whereas the *tube* is an indispensable effector molecule in the Toll pathway. The synergic regulatory mechanism of miR-959-962 cluster in the *Drosophila* immune response to *M. luteus* infection could negatively regulate the Toll pathway in combination *via* directly targeting the 3’UTR of the *tube*, *dl*, or *Toll* mRNAs, leading to a reduced survival rate of flies by inhibiting the expression of AMPs at the late stage of the infection (137). The same study further demonstrated that miR-960 might modulate antibacterial defense only at the late 12-h stage upon infection. At the same time, miR-959 may constantly repress the *Dr* expression at 6 and 12 h, respectively. Meanwhile, miR-961 may contribute more than miR-962 to repress antibacterial defense. Furthermore, using *in silico* screening strategy combined with the Gal80*^ts^*-Gal4 driver system, miR-958 was identified as a candidate miRNA that could potentially regulate the Toll pathway signaling, both *in vitro* and *in vivo*, by negatively targeting *Dif* and *Toll*. The latter is specifically and significantly inhibited by miR-958 at its site 3, as the *Toll* 3’UTR harbors four miR-958-binding sites (141). Similarly, *Drosophila* miR-317 negatively regulated *Drosophila* Toll signaling response *via* suppressing only the *Dif*-Rc of *Dif* four isoforms (142). However, the same authors found in a previous study that miR-317 regulates the *Drosophila* Toll pathway *via* targeting the three other *Dif* isoforms (*Dif-Ra/b/d*) (141). A set of studies showed the involvement of miR-317 in *Drosophila* reproductive responses and larval ovary morphogenesis (143–145). Remarkably, flies transiently overexpressing miR-317 have poor survival. In contrast, the knockout miR-317 flies (miR-317 KO/+) have better survival than the control group, respectively, during Gram-positive bacterial infection (142), implying a new insight of miRNA involved in the crosstalk between *Drosophila* immune and survival. Moreover, four members of the *Drosophila* miR-310, namely miR-310, miR-311, miR-312, and miR-313, negatively regulated the Toll-mediated immune response by repressing the expression of *Drs* and directly cotargeting the 3’UTR of *Drs* in *Drosophila* upon Gram-positive bacterial infection (146). In summary, insect-encoded miRNAs can act individually or collectively to inhibit AMP expression and impair antibacterial defenses for the purpose of immune homeostasis. These mechanisms, in most cases, involve the regulation of the Toll pathway components and allow not only the identification of a new miRNAs but also enrich the repertoire of Toll-related immune-modulating miRNAs in insects.

While some insects enjoy the conservation of genes encoding immune effectors, other from the hemipteran species such as pea aphid *Acyrthosiphon pisum* showed decreased immune responses as they lack the genes coding for AMP, IMD, PGRPs, and other immune-related molecules (53). Fortunately, the conserved Jun N-terminal kinase (JNK) pathway has been suggested in the pea aphid immune defense. In their investigation on how this pathway regulates the pea aphid immune defense upon bacterial invasion, Ma et al. (147) found that miRNA-184a/b targeted the JNK-3’UTR and repressed its expression, hence resulting in more bacteria in the aphids and increased aphids’ mortality after infection. The expression of miRNA-184a and miRNA-184b remarkably dropped after *M. luteus* and *Pseudomonas aeruginosa* infection, with the lowest expression observed at 24 h postinfection, revealing a negative correlation with JNK expression (147). Interestingly, PO, reactive oxygen species, and phagocytosis are under the control of the JNK pathway, suggesting miRNA-184 indirectly controls these antibacterial immune response mechanisms in the pea aphid. Finally, the regulation of the JNK pathway by miRNA-184 is likely a universal mechanism in animals, as prediction using the RNAhybrid program showed that JNK is a potential target of miRNA-184 in insects, zebrafish, frogs, mice, and humans (147).

UPEC strains provoke symptomatic urinary tract infections in humans, whereas commensal-like *E. coli* strains in the urinary bladder cause long-term asymptomatic bacteriuria (ABU). *G. mellonella* is a surrogate insect model host used to study human pathogens, including UPEC (148). miRNA sequencing in *G. mellonella* larvae infected with UPEC strain CFT073 or ABU strain 83972 showed significant changes in the expression levels of *G. mellonella* miRNAs, respectively, suggesting that insect immune response-mediated miRNAs can distinguish between pathogenic and commensal *E. coli* invasions (136).

#### 4.1.3 Upon Viral Invasion

Host miRNAs have an essential role in defense against viral attacks, hence influencing the course of the infection. For example, during chikungunya virus (CHIKV) infection of *A. aegypti*, upregulated *A. aegypti* miR-2b binds to the ubiquitin-related modifier (Urm) 3’UTR, decreasing its translation. This finally leads to decreasing CHIKV replication within *A. aegypti* (26).

In contrast, in some cases, insect miRNAs can promote viral replication by reducing the expression of virus-induced host genes. That is the case for the insulin-related peptide-binding protein 2 (IBP2) which is known to be significantly upregulated in viruses- infected B. mori (149) but has been negatively regulated by miR-278-3p in vitro and vivo, leading to *BmCPV* replication. However, the definite mechanism of miR-278-3p and IPB2 on *BmCPV* replication has been ambiguous, requiring further investigation in the future (150).

### 4.2 Role of lncRNAs in Insect Immunity Upon Microbial Invasions

**Figure 5:**
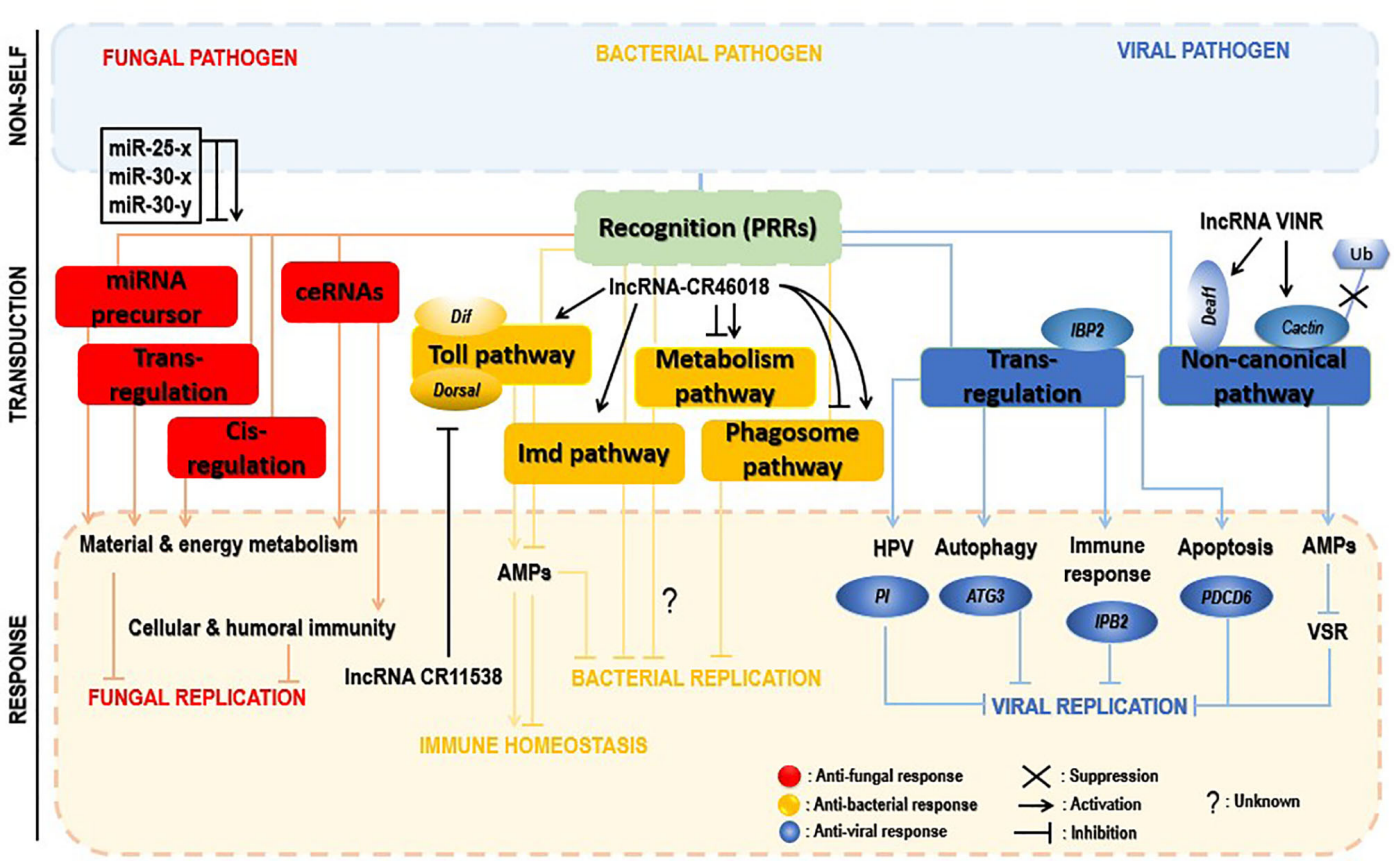
Insect immune defense modulation by lncRNAs upon microbial attacks. The fungal invasion of insects induced alteration of a myriad of insect lncRNAs, which regulate neighboring genes in *cis* and *trans* or act by interacting with miRNAs (sponges) or being miRNA precursors. Those *trans*-and *cis-acting* predominantly activate material and energy metabolism processes and cellular and humoral immunity, hence helping in control of the infection (red). lncRNAs inhibit bacterial replication *via* positive regulation of the Toll pathway, the phagosome pathway, or the metabolism process. However, the latter signaling pathway required more investigation to understand how its deactivation contributes to decreased pathogen replication. Additionally, to avoid immune overactivation after bacterial invasion, insect lncRNAs decoy the critical components of the Toll pathway, lowering the expression of AMPs, thus promoting host immune response homeostasis (yellow). During the viral invasion of insects, insect lncRNAs positively *trans*-regulate insect genes (PI, ATG3, IBP2, PDC6, etc.) involved in cellular and humoral immune-related pathways (HPV, autophagy, apoptosis, etc.). Viral suppression is also achieved through activation of a noncanonical pathway, as an alternative to compensate the RNAi pathway failure; deployed lncRNAs inhibit both the virulence suppressor of RNAi (VSR) and the ubiquitination of cactin in the nucleus or indirectly target the transcription factor Deaf1 and the RNA polymerase II (RNAPII) for transcription of AMPs to control the viral replication (blue).

The functional exploration of lncRNAs in insects is by far exhaustive, mainly upon microbial attacks. **Figure 5** gives an overview of how insects use lncRNAs to defend against fungal, bacterial, or viral attacks.

#### 4.2.1 Upon Fungal Invasion

Few reports investigated interactions between insects and fungi, mainly insect lncRNA response to fungal stress. The western honeybee *Apis mellifera* (*A. mellifera*) is domestically used worldwide for honey production and crop pollination. The spore-forming and obligate intracellular fungal pathogen, *Nosema ceranae* (*N. ceranae*), can infect a variety of insects, including honeybees (151). The expression of *A. mellifera* lncRNAs was drastically altered by *N. ceranae* infection, revealing 4,749 conserved lncRNAs and 1,604 novel lncRNAs. Some differentially expressed lncRNAs regulated gene expression in *cis* and *trans* manners or served as precursors of miRNAs, or competitive endogenous RNAs (ceRNAs) acting as miRNA sponges, leading to the activation of the host key signaling pathways and infection control (152). One of the essential suggestions was that through their sponge potential, *A. mellifera* differential expressed lncRNAs might suppress *N. ceranae via* crosstalk with miR-25-x, miR-30-x, and miR-30-y. However, the binding of these lncRNAs and miRNAs is limited to bioinformatic prediction, and further experimental study is required.

#### 4.2.2 Upon Bacterial Invasion

LncRNAs can promote or impair insect immune responses, especially the Toll immune response upon bacterial attacks. *Dif* and *Dorsal* genes are two crucial components of the Toll immune signaling in insects. These two effectors can activate the transcription of AMPs for pathogen eradication. The lncRNA CR46018 was approximatively tenfold overexpressed after infection of *Drosophila* with *M. luteus*. In addition, RNA-seq analysis of lncRNA CR46018-overexpressing *Drosophila* after infection with *M. luteus* revealed that upregulated genes were mainly enriched in Toll and Imd signaling pathways, supported by bioinformatics predictions and RNA-immunoprecipitation experiments which showed that CR46018 interacted with the transcription factors Dif and dorsal to enhance the Toll pathway. All this indicates lncRNA-CR46018 as a positive regulator of the Toll signaling pathway and essential for *Drosophila* survival. Moreover, lncRNA-CR46018 could up- and downregulate genes involved in the phagosome pathway and metabolism-related regulation, respectively. The latter pathway seems to be a promising target of insect-derived lncRNAs during insect–pathogen interactions. For example, a previous report unveiled that the *Drosophila* lncRNA CR44404 (lincRNA-IBIN) links immunity and metabolism in *Drosophila* upon infection with *M. luteus* (28). However, how insect-derived lncRNAs regulate this pathway to support insect immune response mechanisms needs further investigation.

Inversely, lncRNAs could negatively regulate insect immune responses by suppressing insect immune effectors to avoid abusive immune stimulation at a late stage of the infection. A recent study reports that at the later stage (24 h) of infection of *Drosophila* with *M. luteus*, the lncRNA-CR11538 inhibited the transcription of AMPs *via* decoying Dif/dorsal away from AMP promoter, thereby negatively modulating the Toll signaling pathway and inhibiting their transcriptions to prevent abusive immune activation in *Drosophila* with *M. luteus* infection (153).

#### 4.2.3 Upon Viral Invasion

The RNAi pathway is an essential antiviral response in insects. However, several studies suggested that the RNAi pathway is ineffective in preventing viral replication (154, 155). An obvious viral strategy to bypass insect RNAi defense mechanisms is to encode RNAi suppressors. Several RNAi cancellers have been described in plant and insect RNA viruses (156, 157). Meanwhile, no RNAi suppressors have been associated with arboviruses until the recent investigation of Zhang et al. (158). During infection of *Drosophila* with the *Drosophila* C virus (DCV), the antiviral lncRNA VINR was accumulated in the nucleus because of DCV’s viral RNAi suppressors for their inhibition. LncRNA VINR acted by binding to cactin, preventing its degradation by ubiquitin-proteasome and promoting a noncanonical antiviral and AMP defense leading to a reduced viral replication (159). This suggests a counter counter-defense strategy activated by a lncRNA in response to the viral suppression of the primary antiviral RNAi immunity in *Drosophila*.

LncRNAs regulate gene expression *via cis*- or *trans*-acting regulation (160, 161). In a comprehensive study of lncRNAs associated with Rice black-streaked dwarf virus (RBSDV) infection in *Laodelphax striatellus* midgut, all predicted and differentially expressed mRNA targets were regulated in a *trans* manner by 176 differentially expressed lncRNAs. In addition, although the KEGG pathway analysis revealed significantly enriched pathways such as purine metabolism, valine, leucine, and isoleucine degradation, fatty acid elongation, and so on as the most significantly enriched pathways of those trans-regulated genes, the fact remains that the Human papillomavirus infection pathway (which is pivotal for viral infection) was enriched considerably during RBSDV infection. It, therefore, might be involved in RBSDV infection of *L. striatellus* midgut (138). The eight differentially expressed lncRNAs and the two coexpressed targets in the Human papillomavirus pathway predicted by KEGG analysis and confirmed by RT-qPCR can be found in **Table 1**. Moreover, a protease inhibitor (PI), one of the lncRNAs’ targets, plays a vital role in antivirus and preventing carcinogenesis (162, 163). Interestingly, lncRNA MSTRG15394 and its target PI (15-fold) were significantly expressed, and scrutinizing their involvement in RBSDV infection of *L. striatellus* midgut showed that knockdown of MSTRG15394 or PI drastically increased the expression patterns of RBSDV replication-related genes, S5-1, S6, and S9-1, suggesting that MSTRG15394 and PI could inhibit the accumulation and proliferation of RBSDV in *L. striatellus* midgut (138). Moreover, upon *B. mori* cypovirus (*BmCPV*) infection of silkworm larvae, the expression of mRNA targets was mainly affected *via trans*-regulation by *BmCPV*-induced lncRNAs (164). Interestingly and remarkably, analysis of the differentially expressed lncRNAs and mRNAs network reveals that these differentially expressed lncRNAs simultaneously targeted some genes involved in relevant mechanisms such as apoptosis (PDCD6), autophagy (ATG3), and immune response (IPB2). Genes such as PDCD6, ATG3, IPB2, MFB1, and VPS52 could be *trans*-targeted by the most significantly expressed lncRNA MSTRG.20486.1 (164).

## 5 Pathogen-Encoded ncRNAs Modulating Insect Immunity

Pathogens can encounter insect immune responses and create a suitable environment for their replication. Insect pathogens have employed several strategies to escape host immune responses, including pathogen-encoded miRNAs and lncRNAs. The pathogen-encoded ncRNAs targeting insect-derived genes are listed in **Table 3**.

**Table 3:** Pathogen-encoded ncRNAs targeting insect-derived genes.

Pathogen	miRNA	Target	Expression level	Host target	Ref.
*B. bassiana*	bba-milR1	*Spz4*	Down	*A. stephensi*	(158)
*B. bassiana*	bba-milR1	*CLIPB9*	Up	*A. stephensi*	(158)
*N. bombycis*	Nb-milR8	*BmPXE16*	Down	*B. mori*	(165)
*BmCPV*	BmCPV-miR1	*BmRan*	Down	*B. mori*	(166)
*BmCPV*	BmCPV-miR3	*BmRan*	Down	*B. mori*	(166)
*BmCPV*	BmCPV-miR-10	*BmCSDE1*	Down	*B. mori*	(167)
*BmCPV*	BmCPV-miR-10	*BmApaf-1*	Down	*B. mori*	(167)
*BmCPV*	BmCPV-miR-1	*BmIAP*	Down	*B. mori*	(168)

### 5.1 Pathogen-Encoded miRNAs Modulating Insect Immunity

**Figure 6:**
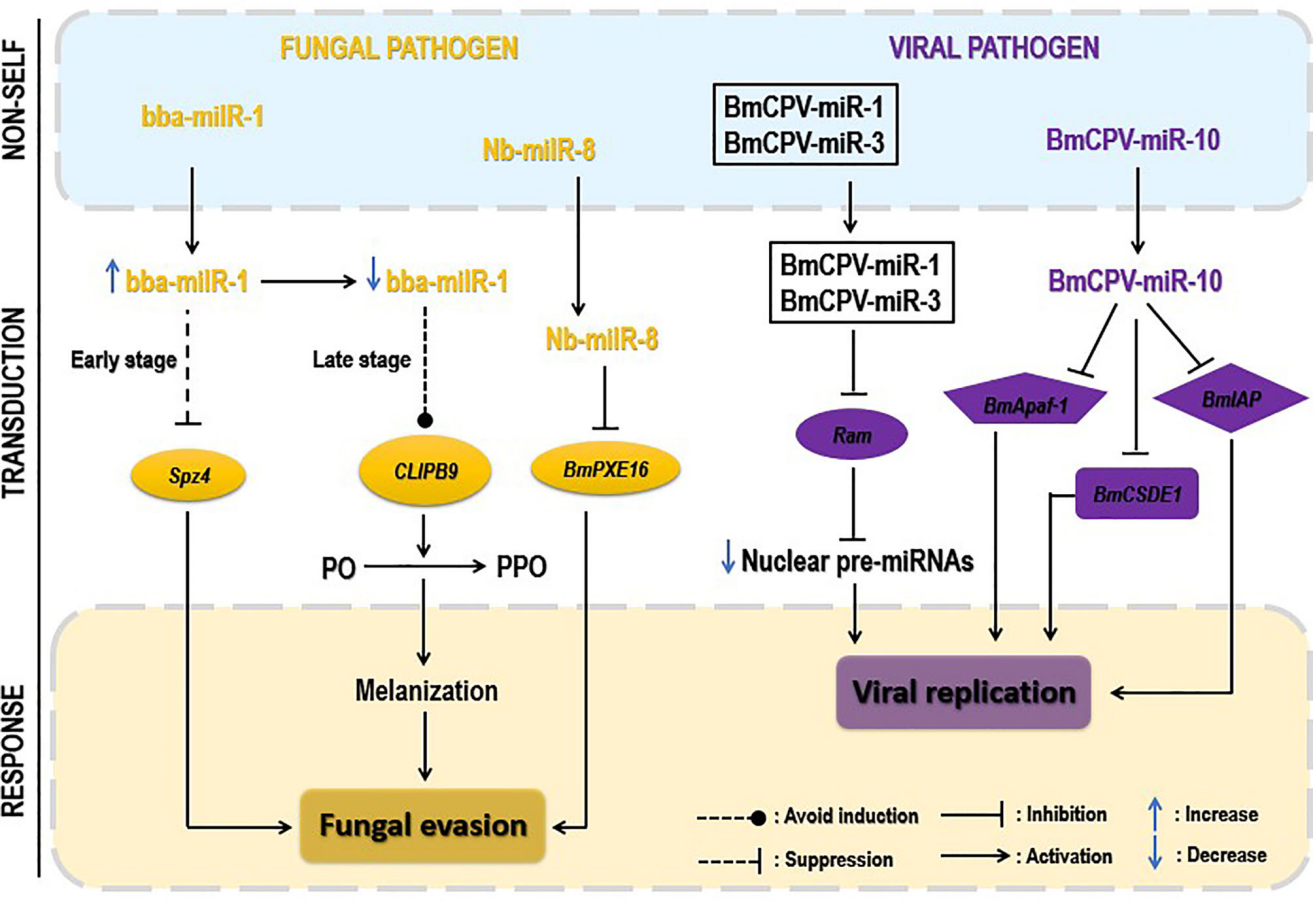
Pathogen-encoded miRNAs regulating insect immunity. Pathogen-derived miRNAs often translocate *via* extracellular vesicles (EVs) to regulate insect host immunity. Upon fungal invasion, translocated fungal miRNAs downregulate the insect Toll signaling pathway by repressing the expression of critical genes, such as *Spz4*, or inhibiting the peroxisome pathway, repressing the expression of the PXE16 gene. At the late stage of infection, fungi act by decreasing the expression of their miRNAs to escape the melanization process (yellow). Upon viral attacks, translocated virus-derived miRNAs act individually or synergistically to negatively regulate the expression of insect key genes (Ran, Apaf-1, etc.) by targeting their 3′UTR regions. The latter downregulation reduces host miRNAs’ production, leading to viral replication and proliferation (purple). However, the figure does not show the bacterial-derived miRNAs due to missing data on their role in insect–bacterium interaction.

Pathogen-derived miRNAs or miRNA-like-RNAs (milRNAs) are necessary components for host–pathogen crosstalk, promoting pathogen proliferation and replication in most cases. These milRNAs can modulate both pathogen and insect host immune genes. The following concentrates on the milRNA modulation of insect key genes. **Figure 6** highlights the manipulation of insect immunity by pathogen-encoded miRNAs.

#### 5.1.1 Fungus-Encoded miRNAs

milRNAs play crucial roles during fungal invasion (169). However, few fungal-derived milRNAs have been reported. Still, their active investigation remains challenging.

Delivering cell-entering effector molecules is a well-known employed strategy of plant pathogenic fungi to suppress host immunity (170, 171). No description has been documented for such effector molecules for pathogenic insect fungi until the brilliant discovery of Cui et al. (158). The pathogenic fungal *B. bassiana* deployed a miRNA-like RNA (bba-milR1) acting as a cell-entering effector to suppress mosquito immune response. The authors found that during the early stages of infection, *B. bassiana* bba-milR1 expression increased remarkably and translocated into the mosquito cells to mitigate mosquito immune responses by suppressing the expression of the critical activator gene *Spz4*. During this early invasion in the integument, the bba-milR1 is not accessible to circulating hemocytes, the site of CLIPB9 gene expression. In contrast, during the late stage of infection (hemocoel invasion), *B. bassiana* strategically decreased the expression level of bba-milR1 and avoided induction of CLIPB9 and activation of melanization. Quantification of bba-milR1 expression level during *B. bassiana* infection of *Anopheles stephensi* (*A. stephensi*) showed that bba-milR1 was induced by ~30-fold at 36 h postinfection and then declined to deficient levels as the fungus enters the host’s hemocoel at about 60 h after infection (158). Moreover, *Nosema bombycis* (*N. bombycis*) proliferation within *B. mori* is mediated by the increased expression of its miRNA-like RNA, Nb-milR8, which negatively regulates the *B. mori* peroxisome metabolic pathway *via BmPXE16* gene expression, ultimately inhibiting the latter expression (165).

#### 5.1.2 Virus-Encoded miRNAs

Studies have shown that one miRNA can target multiple genes and several miRNAs can also regulate a target gene. Generally, miRNA mainly binds to the 3’UTR of mRNA to repress the target gene translation, and cooperativity between two or more miRNA-binding sites can enhance the repression of the mRNA translation (172–174). In the case of viral infections, virus-encoded miRNAs generally target the 3’UTR of key insect immune genes to repress their expressional changes and reduce the generation of insect miRNAs to create a favorable environment for viral replication (175). Lin et al. (166), for example, reported that to facilitate its replication in *B. mori*, *BmCPV* released two miRNAs, BmCPV-miR-1 and BmCPV-miR-3, that co-operatively targeted the 3’UTR of *B. mori* GTP-biding nuclear protein Ran (BmRan) by lowering its expression level. Ran is a GTP-binding nuclear protein of 25 kDa, which plays a role of transporting small RNAs, including pre-miRNAs, from the nucleus to the cytoplasm (176), and its repression level led to a significant reduction in nuclear export of pre-miRNA. Similarly, BmCPV-miR-10 downregulated the *B. mori* cold shock domain E1 protein (BmCSDE1) mRNA expression level after binding to its 3’UTR in the infected larvae. BmCPV-miR-10 also inhibited the expressional changes of *B. mori* apoptotic protease activating factor 1 (BmApaf-1) during *in vivo* infection, creating suitable conditions for virus replication and proliferation (167). BmCPV-miR-1 inhibited cell apoptosis in the infected silkworm *via* increasing *B. mori* inhibitor of apoptosis protein (BmIAP) expression, promoting the virus replication (168). Obviously, the roles of virus-encoded miRNAs in the crosstalk seem to be largely explored compared with miRNA-encoded fungi or bacteria. An interesting insight in investigating this element-encoded virus is that they have the possibility to simultaneously regulate both insect innate and cellular immune responses, and further demonstrate that there is a dynamic evolution in the insect–pathogen crosstalk implicating ncRNAs, where insects evolutionally sophisticate their defense mechanisms while pathogens simultaneously elaborate strategies to encompass these immune obstacles for their own benefit.

### 5.2 Pathogen-Encoded lncRNAs Modulating Insect Immunity

Although new technologies and bioinformatics tools emerge for the detection of ncRNAs, most lncRNAs’ expression is low, probably limiting their identification and functional characterization (177). Several studies identified pathogen-encoded lncRNAs, their differential expression levels, and how the latter regulates pathogen’s genes (178). However, studies describing how these pathogen-encoded lncRNAs regulate insect immune responses during insect–pathogen interactions are considerably missing. For example, no virus-encoded lncRNAs were generated during silkworm larvae-*BmCPV* interaction, while 41 *B. mori* lncRNAs among 1845 were identified (164). We are tempted to speculate that pathogen-encoded lncRNAs may act similarly to miRNAs derived-pathogens, where their differential expression negatively regulates insect key genes and creates a favorable environment for pathogen replication and proliferation. However, the above suggestion needs further experimental shreds of evidence. This area could be an exciting and hotspot topic for researchers to investigate in the future.

## 6 Conclusions and Perspectives

ncRNAs are the emerging and fate-determining players of insect–pathogen interactions. The long history of host and pathogen coevolution suggests that the pathogen keeps on evolving its arsenals to succeed in infection, whereas the host continues investing in defense mechanisms. Therefore, portraying the landscape of ncRNAs in insect–pathogen crosstalk at the immunological level is of great scientific worth. In short, we briefly reviewed the main signaling pathways and accessory immune mechanisms engaged in insect defense mechanisms and the recent progress on the involvement of ncRNAs, especially miRNAs and lncRNAs, in promoting or suppressing insect immune responses upon pathogen invasion. In addition, how pathogen-encoded miRNAs and lncRNAs regulate insect immunity was a part of the scope of this study.

The Toll, Imd, and JAK/STAT pathways are evolutionarily conserved pathways playing a manifest role in insect immune defense mechanisms against pathogens. They protect a wide range of pathogens (bacteria, fungi, parasites, etc.). In addition, with the assistance of immune effector mechanisms, this protection might be strengthened. However, looking deep inside, although the conferred protection, several factors exclude the idea of total protection. The fact that these pathways are not present in all insects might influence and change the ability of insects to defend themselves upon pathogens invasions. Moreover, the inexistence of adaptive immunity, which confers immune memory, allowing organisms to mount a more rapid and potent immune response when it is re-exposed to the same pathogen or a highly related one, might probably be a situation that affects insect immune defense intensity and efficacy. However, it has been hypothesized that immune priming occurs because the insect is inherently able to activate immune responses more rapidly during a second exposure or because pathogens or pathogen components are retained within the insect, which maintains the animal in a heightened state of immune alertness (179).

Insect immunity is well known to be modulated by several factors, including microbiota and ncRNAs. The latter, mainly miRNAs and lncRNAs, hugely reinforce insect host immune defense mechanisms during their crosstalk with pathogens. Their positive and negative modulation of insects’ immune responses passes through their ability to target insect- or pathogen-encoded mRNA targets and influence the course of the infection for the benefice of insects. These elements can be viewed as alternative insects’ defense mechanisms such as those mentioned above (immune effector mechanisms). They can be put on the stage as primary insect immune defense mechanisms, when principal pathways are inefficient to secure insects’ integrity from being invaded. The RNAi immune effector mechanism, for example, is well documented to provide a robust protection to insects against viral attacks. However, pathogen VSRs were recently proved to overcome this path (180). In this present case, the insect’s survival was accountable to the immune modulation ability of ncRNAs. Therefore, it is hoped that these noncoding elements would be exploited as a mainstream player to achieve food security or avoid economic waste in the industry by tackling different insect invaders.

Although their ability to maintain and restore insect immune homeostasis or enhance host immune defenses, ncRNA protection seems to be limited as their downregulation action of insect immunity promotes, in some cases, the proliferation of the pathogen. Interestingly, in opposition to pathogen-derived miRNAs, there is a deficiency of studies describing the modulation of insect immunity by pathogen-derived lncRNAs in order to facilitate pathogen replication and proliferation. Such topics should retain researchers’ attention, as exploring this area will undoubtedly bring new insights into the role of these elements in insect–pathogen crosstalk. In addition, it seems like ncRNAs support insect immunity against pathogens by regulating insect main and evolutionarily conserved signaling pathways (Toll, Imd). Simultaneously, their action is less observed in insect accessory immune mechanisms. The regulation of these insect accessory defense mechanisms by ncRNAs has not been extensively explored. Few studies investigated the link between ncRNAs and insect accessory defense mechanisms. miRNAs of *Snellenius manilae bracovirus* (SmBV-miR-199b5p and SmBV-miR-2989) were found to regulate innate (*domeless* and *toll-7*) and cellular (encapsulation and phagocytosis activity of the hemocytes) immune responses of its host *Spodoptera litura* (181). *Plutella xylostella* miR-8 is downregulated following parasitization by *Diadegma semiclausum*, leading to significant declines in Serpin 27 transcript levels (182). The serine protease inhibitor Serpin 27 regulates activation of the Toll pathway and PPO involved in the melanization response in insects. Taken together, we are tempted to speculate that there is no direct role of ncRNAs on insect accessory immunity. Nonetheless, to a lesser extent, and based on evidence, ncRNAs undoubtedly modulate insect immune effector mechanisms indirectly by targeting insect main signaling pathway components. The Toll ligand *Spätzle 3* controlled the melanization process in the stripe pattern formation of caterpillars (183).

It is now known that ncRNAs have a drastic immunomodulation influence in insect–pathogen crosstalk. All the changes, such as the differentially expressed miRNAs, lncRNAs, the co/target transcripts, and the diverse signaling pathways activated during insect–pathogen crosstalk, are proof of their crucial role. Proper understanding of insect–pathogen interaction is essential to deciphering the immune molecular patterns employed by insects against various pathogens. This understanding will positively impact the economy based on important insects, such as *D. melanogaster* and *B. mori*. Here are some perspectives: (1) improvement of methods and technologies used for ncRNA investigations; (2) promotion of more in-depth studies instead of preliminary ones; and (3) the link between pathogen-encoded lncRNAs and insect immunity represents an attracting topic to investigate and may hide tons of critical information, which will undoubtedly give new insights into the immunological landscape of insect–pathogen interaction.

## Author Contributions

UAEM and TT reviewed the literature, designed the figures, and wrote the manuscript. LS, GY, and XL designed the tables and reviewed the manuscript. ZS, YW, and HC reviewed and revised the text. All authors listed have made a substantial, direct, and intellectual contribution to the work and approved it for publication.

## Funding

This work was supported by the National Natural Science Foundation of China (31802142), the Doctoral Start-up Fund of Southwest University (SWU120019, SWU020023), the Fundamental Research Funds for the Central Universities (XDJK2019C089), and the China Postdoctoral Science Foundation (2019T120801 and 2017M620408).

## Conflict of Interest

The authors declare that the research was conducted in the absence of any commercial or financial relationships that could be construed as a potential conflict of interest.

## Publisher’s Note

All claims expressed in this article are solely those of the authors and do not necessarily represent those of their affiliated organizations, or those of the publisher, the editors and the reviewers. Any product that may be evaluated in this article, or claim that may be made by its manufacturer, is not guaranteed or endorsed by the publisher.
